# Phytochemical Profiling and Computational Screening of *Musa acuminata* Peel as Hemorrhagic Wound Treatment Candidate: Network Pharmacology, Molecular Docking, Molecular Dynamics, and DFT Approaches

**DOI:** 10.3390/ph19070992

**Published:** 2026-06-26

**Authors:** Andi Darma Putra, Naufal Syafiq Darmawan, Lasmini Syariatin, Aldi Tamara Rahman, Edwin Jeika Bunggulawa, Firda Puspita

**Affiliations:** 1 Division of Gynecologic Oncology, Department of Obstetrics and Gynecology, Faculty of Medicine, Universitas Indonesia, Cipto Mangunkusumo Hospital, Central Jakarta 10430, Indonesia; 2 Ovarian, Tubal, and Peritoneal Malignancy Research Unit, Department of Obstetrics and Gynecology, Faculty of Medicine, Universitas Indonesia, Cipto Mangunkusumo Hospital, Central Jakarta 10430, Indonesia; naufal.syafiq71@ui.ac.id (N.S.D.); lasminisyariatin@mail.ugm.ac.id (L.S.); 2010422018_aldi@student.unand.ac.id (A.T.R.);; 3 Dopamine Science Institute, Depok 16431, Indonesia; 4 Department of Pharmacy, Faculty of Health Sciences, Universitas Pelita Harapan, Tangerang 15811, Indonesia; edwin.bunggulawa@uph.edu

**Keywords:** *Musa acuminata*, hemorrhagic wound healing, network pharmacology, molecular docking, natural product-based drug discovery

## Abstract

**Background:** Hemorrhagic wounds pose significant clinical challenges, with approximately 20% associated with surgical site infections and an increased mortality risk. Despite growing interest in natural product-based medicines, the molecular targets and bioactive phytochemicals of *Musa acuminata* peel relevant to hemorrhagic wound healing are insufficiently established. **Methods:** This study employed an integrative in silico approach to identify bioactive phytochemicals from the ethyl acetate extract of *Musa acuminata* peel as potential wound healing agents. Liquid chromatography-high resolution mass spectrometry (LC-HRMS) profiling was performed for phytochemical characterization, followed by drug-likeness and toxicity screening via OSIRIS DataWarrior. Network pharmacology, molecular docking, molecular dynamics (MD), binding free energy calculation, pharmacokinetic properties prediction, and density functional theory (DFT) analysis were subsequently conducted. **Results:** LC–HRMS profiling identified 211 compounds across 21 chemical classes, of which 18 met drug-likeness criteria. Network pharmacology revealed five key protein targets. Molecular docking demonstrated that **Compound 16** (−9.34 kcal/mol) and **Compound 17** (−9.26 kcal/mol) exhibited stronger binding affinity toward VEGFR2 than Axitinib (−9.15 kcal/mol), with key interactions at glutamic acid-917 (GLU917) and cysteine-919 (CYS919). MD simulations over 100 ns confirmed complex stability, with **BP16** showing superior binding stability and favorable MM/PBSA free energy. Pharmacokinetics and DFT analysis further supported **BP16** as the most promising lead compound, exhibiting favorable pharmacokinetic properties, low predicted toxicity, and enhanced electronic stability. **Conclusions: BP16** and **BP17** are identified as potential VEGFR2-targeting candidates, providing a rational mechanistic foundation for future experimental validation as natural hemorrhagic wound healing therapeutics.

## 1. Introduction

The skin is the body’s largest organ, serving as a key physical and chemical barrier against external trauma, microbial pathogens, and mechanical injury [1,2]. Maintaining the structural integrity of the skin is essential for physiological health. Disruption of this barrier, particularly caused by traumatic injury, can result in hemorrhagic wounds that, if not properly treated, significantly increase the risk of infection, sepsis, chronic wound formation, and mortality [3,4,5]. Epidemiological data indicate that approximately 20% of hemorrhagic wounds are complicated by surgical site infections, of which more than 40% progress to sepsis [6,7,8]. Moreover, hemorrhagic wounds are independently associated with an eleven-fold increase in mortality risk, highlighting the urgent clinical demand for effective wound healing treatments [9].

Conventional wound healing agents, including sterile gauze, film, and hydrocolloid, remain among the most widely used wound care modalities, owing to their ability to prevent external contamination, absorb wound exudate, and maintain a moist microenvironment favorable to tissue regeneration [10,11]. Over time, these materials have undergone advancements through the integration with bioactive excipients, such as silver nanoparticles (AgNPs), growth factors, and stem cells, which have demonstrated potential in improving their therapeutic efficacy [12,13,14,15]. However, several limitations persist, such as high production cost, considerable cytotoxic potential, and susceptibility to denaturation under physiological conditions [16]. Historically, natural products have been the basis of wound care treatments globally, and their resurgence in modern pharmaceutical research has been driven by the disadvantages of synthetic alternatives. As a result, molecules derived from natural products have attracted growing interest as potential therapeutic alternatives due to their superior biocompatibility, ability for multi-targeted pharmacological actions, and relative cost-effectiveness [17].

*Musa acuminata* (Cavendish banana) is among the most widely cultivated tropical fruits globally and is reported to be rich in phytochemicals throughout its components, comprising the peel, pulp, and stem. In particular, the peel is enriched with polyphenols, flavonoids, and biogenic amines, including gallic acid, catechin, ferulic acid, serotonin, and dopamine, which have been reported to confer anti-inflammatory properties [18]. Among these, trigonelline and isovanillic acid have been demonstrated to inhibit bacterial enzymes and suppress key pro-inflammatory signaling cascades, including the tumor necrosis factor-alpha (TNF-α) pathway [19]. Additionally, flavonoids and tannins present in banana peel have been reported to exert vasoconstrictive effects, thus promoting wound closure [20]. Building upon these findings, Rizka et al. (2023) developed an ethanolic banana peels extract-based spray gel for burn wound treatment, exhibiting efficacy against *Escherichia coli* and *Staphylococcus aureus*, with a 38% improvement in wound closure rate compared to the control group [21]. However, existing research on *M. acuminata* peel has mainly focused on crude extract bioactivity and formulation, without systematically identifying the active phytochemicals or their mechanisms. No studies have specifically explored these compounds in hemorrhagic wound healing at the molecular level, including their protein targets and signaling pathways. Therefore, the pharmacological targets, underlying mechanisms, and key bioactive constituents are still inadequately characterized, which limits the development of therapeutics.

Recent developments of bioinformatics and computational assays have become increasingly essential to drug discovery pipelines, especially for natural product-based therapeutics. This approach assists in target identification, ligand design, and molecular pathways elucidation through approaches such as protein–protein interaction analysis, which enables the identification of affected pathways and potential sites of drug intervention [22]. Moreover, virtual screening and molecular docking provide reliable predictions of ligand binding affinities and configurations, considerably improving the drug design process before experimental synthesis and validation [23]. Due to the complexity of phytochemical mixtures and the multi-targeted characteristics of natural products, integrated in silico methods are particularly suitable for exploring the therapeutic mechanisms of compounds derived from *M. acuminata* peels. To the best of our knowledge, no previous study has utilized an integrated phytochemical profiling and a comprehensive in silico analytical pipeline to systematically characterize the phytochemical composition of *M. acuminata* peel ethyl acetate extract and determine the molecular mechanisms that underlie its hemorrhagic wound healing activity. We propose that specific drug-like phytochemicals in the extract are able to interact with key wound healing targets, exhibiting high binding affinity and stability to serve as promising leads for therapeutic development.

Herein, this study aimed to investigate the therapeutic potential and molecular mechanisms of phytochemicals from *M. acuminata* peel as wound healing agents using an integrated in silico methods. The ethyl acetate extract was subjected to phytochemical profiling through liquid chromatography and high-resolution mass spectrometry (LC-HRMS) to identify the constituent compounds. A comprehensive in silico pipeline was employed, involving virtual screening, network pharmacology, molecular docking, molecular dynamics simulation, and density functional theory (DFT) analysis. By identifying potential lead compounds from *M. acuminata* peel and delineating their molecular targets and mechanisms of action in hemorrhagic wound healing, this study contributes to the growing body of evidence supporting natural product-based drug discovery and provides a rational mechanistic foundation for future experimental validation and therapeutic development.

## 2. Results and Discussion

### 2.1. Phytochemical Profiling of M. acuminata Peel Ethyl Acetate Extract

**Figure 1:**
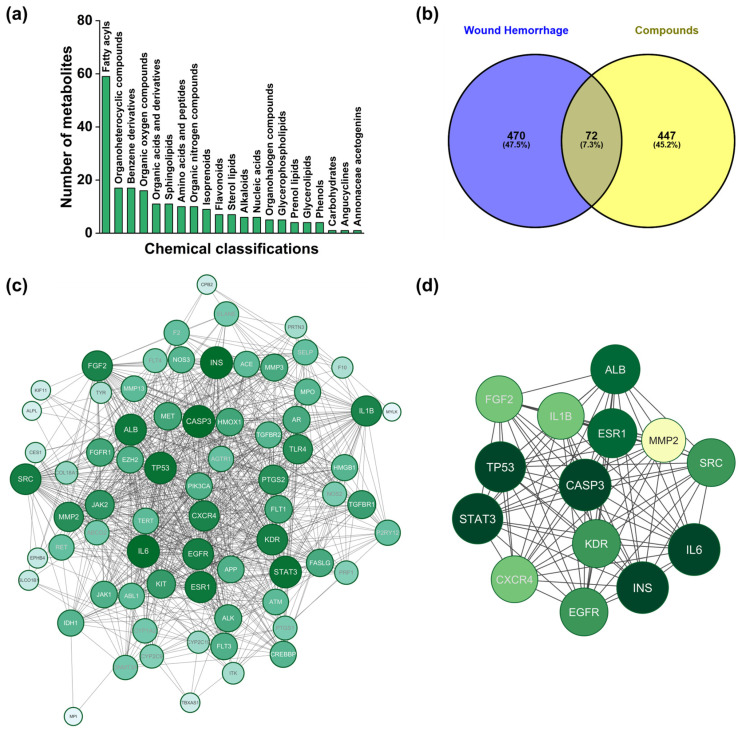
Phytochemical profiling and target protein analysis. (**a**) Classification of 211 compounds identified from *M. acuminata* peel, (**b**) overlapping targets between 18 screened compounds and hemorrhagic wound-related targets, (**c**) the protein–protein interaction (PPI) network of 72 common targets (light turquoise to dark green indicating increasing degree), and (**d**) topological analysis of the PPI network highlighting 14 hub nodes (yellow to dark green indicating increasing degree).

The extraction of *M. acuminata* peel was carried out using the cold maceration technique employing ethyl acetate as the solvent. This method allows selective extraction for semi-polar phytochemicals while preserving their structural integrity and bioactivity [24]. The crude extract was subsequently subjected to LC-HRMS analysis, which identified 211 compounds with fatty acyls representing the most abundant chemical class (26.94%), followed by organoheterocyclic compounds (8.68%), benzene derivatives (7.76%), organic oxygen compounds (7.76%), and organic acids (5.02%) among the top five (Figure 1a; Supplementary Table S1). The prevalence of fatty acyls aligns with their roles in hemostasis and wound healing. They are essential for regulating platelets during hemostasis, particularly in aggregation through Cyclooxygenase 1 (COX-1)-mediated activation [25]. Moreover, fatty acyls, such as omega-3 fatty acids, have been reported to modulate the inflammatory phase of wound healing by promoting early epithelialization, thereby accelerating wound closure [26,27]. Additionally, organoheterocyclic compounds demonstrated several bioactivities associated with wound healing, including reactive oxygen species (ROS) scavenging, antimicrobial effects, and analgesic properties [28]. Collectively, these findings suggest that phytochemicals extracted from *M. acuminata* peel may serve as a potential natural product-based wound healing agent.

### 2.2. Initial Pharmacological Screening and Network Construction

The three-dimensional (3D) structures of 211 *M. acuminata* peel-derived phytochemicals identified by LC-HRMS were retrieved from PubChem (https://pubchem.ncbi.nlm.nih.gov/, accessed on 11 October 2025) web server. Screening process was carried out employing Lipinski’s Rule of Five to select compounds exhibiting acceptable bioavailability, permeability, and drug-likeness. Although this rule was originally developed for oral drug screening, its application to topical and transdermal candidates has been reported as suitable, supporting its significance in wound healing agent candidate screening [29]. Compounds showing no mutagenic, tumorigenic, reproductive, or irritant toxicity properties were also selected. Following this, a total of 18 chosen ligands were used for the target prediction and network pharmacology analysis (Table 1 and Tables S2 and S3).

To investigate the potential therapeutic targets related to the wound healing activity of *M. acuminata* peel phytochemicals, a network pharmacology was constructed based on the intersection of compound-predicted targets and hemorrhagic wound-associated genes. Target prediction of 18 selected phytochemicals resulted in 519 potential targets, while disease target mining via DisGeNET and GeneCards web servers, resulting in the identification of 542 targets. As depicted in Figure 1b, Venn diagram analysis identified 72 overlapping targets, which were classified as the common therapeutic targets for subsequent network construction. A protein–protein interaction (PPI) network was constructed using Cytoscape v3.10.4, comprising 72 nodes and 887 edges (Figure 1c). PPI network analysis is widely employed to understand key signaling interactions between molecular targets, hence enabling the prediction of potential mechanisms of action for drug candidates. This approach is crucial for understanding how the multi-target effects of natural product-derived metabolites converge upon disease-relevant pathways.

Topological parameter filtering of the PPI network identified 14 core PPI hub genes, highly connected nodes likely serving central regulatory roles in hemorrhagic wound healing, namely *CASP3*, *INS*, *IL6*, *P53*, *STAT3*, *ALB*, *ESR1*, *KDR*, *EGFR*, *SRC*, *FGF2*, *IL1B*, *CXCR4*, and *MMP2* (Figure 1d). These hub genes collectively participate in multiple phases of the wound healing cascade. During inflammatory phase, Interleukin 6 (IL6), secreted by macrophages at the wound site, activates the Janus Kinase/Signal Transducer and Activator of Transcription 3 (JAK/STAT3) signaling pathway along with STAT3, thereby promoting proliferation and tissue regeneration [30]. In the proliferative phase, Epidermal Growth Factor Receptor (EGFR) and Proto-oncogene tyrosine-protein kinase Src (SRC) contribute to the Phosphatidylinositol 3′-Kinase/Protein Kinase B (PI3K/AKT) pathway, promoting epithelial cell migration and the initial differentiation of keratinocytes. Notably, SRC phosphorylates EGFR at Tyrosine-845 preceding PI3K/AKT pathway activation [31]. Simultaneously, Vascular Endothelial Growth Factor Receptor-2 (KDR/VEGFR2) and Fibroblast Growth Factor-2 (FGF2) both play pivotal roles in angiogenesis and neovascularization, collectively promoting tissue remodeling during the remodeling phase [32,33]. Matrix Metalloproteinase 2 (MMP2) further supports this process by allowing fibroblast migration to promote neovascularization and extracellular matrix (ECM) remodeling [34].

### 2.3. GO Enrichment and KEGG Pathway Analysis

**Figure 2:**
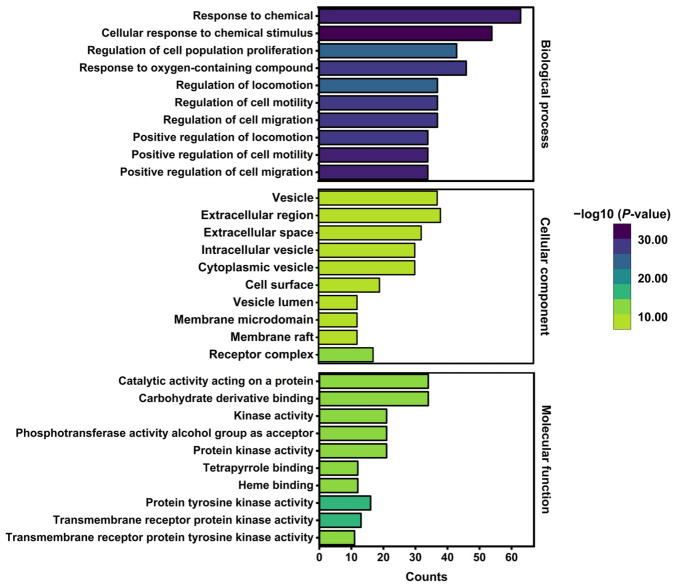
GO enrichment analysis of overlapping targets, including biological processes, cellular components, and molecular functions.

Gene ontology (GO) and Kyoto Encyclopedia of Genes and Genomes (KEGG) pathway analysis were performed to investigate the functional roles of the 72 overlapping protein targets and to identify the signaling pathways most relevant to hemorrhagic wound healing. In this study, GO enrichment analysis resulted in a total of 1000 biological processes, 159 cellular components, and 400 molecular functions, from which the top 10 most significantly enriched terms per category were selected from the false discovery rate (FDR) cutoff value of 0.05 (Figure 2). With respect to biological processes, the target proteins were mainly enriched in response to chemical stimulus, regulation of cell proliferation, and response to oxygen-containing compound, which are collectively implicated in the inflammatory and proliferative phases of wound healing. In terms of cellular components, the target was predominantly localized in vesicles, extracellular region, and extracellular space, suggesting their involvement in paracrine and intercellular signaling. The main molecular functions identified were protein catalytic activity, carbohydrate derivative binding, and kinase activity, associated with the regulatory roles of these targets in intracellular signaling pathways relevant to tissue repair.

**Figure 3:**
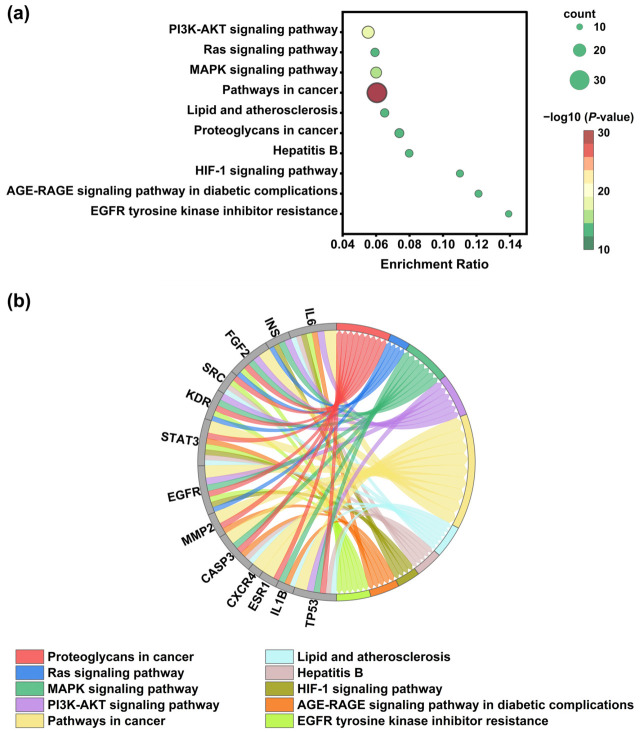
Pathway enrichment analysis related to hemorrhagic wound healing. (**a**) Bubble plot of KEGG pathway enrichment showing the top 10 pathways and (**b**) network depicting the relationship between enriched pathways and 14 hub nodes.

KEGG pathway enrichment analysis identified 203 signaling pathways associated with the 72 common protein targets, of which the PI3K/AKT, MAPK, Ras, and Hypoxia-inducible Factor 1 (HIF-1) signaling pathways are related to hemorrhagic wound healing (Figure 3a). The PI3K/AKT pathway is essential as a central mediator of the proliferative phase of wound healing, facilitating cell proliferation, migration, and the differentiation of keratinocytes [35]. PI3K/AKT engages in regulatory crosstalk with the MAPK pathway to maintain vascular homeostasis during this phase, particularly AKT-mediated downregulation of MAPK signaling reduces pro-inflammatory cytokine responses, including TNF-α, IL6, and IL1β, thereby improving neovascularization [36,37]. Upstream of MAPK, Ras activation initiates signaling cascades that further stimulate keratinocytes migration and angiogenesis, reinforcing the proliferative and remodeling phases of wound repair [38]. Simultaneously, HIF-1 signaling is essential for neovascularization through increasing VEGF secretion, which promotes rapid wound closure under hypoxic conditions [39]. Collectively, the enrichment of these interconnected pathways highlights the multi-targeted pharmacological potential of *M. acuminata* peel phytochemicals in influencing the essential molecular mechanisms involved in hemorrhagic wound healing. To ensure the biological relevance of target selection, pathway filtering was performed based on direct involvement in wound healing physiology, specifically, PI3K/AKT, MAPK, HIF-1, and Ras signaling pathways. Pathways primarily associated with other biological processes, including pathways in cancer, lipid and atherosclerosis, proteoglycans in cancer, Hepatitis B, Advanced Glycation End Products–Receptor for Advanced Glycation End Products (AGE-RAGE) signaling pathway, and EGFR tyrosine kinase inhibitor resistance, were excluded to maintain wound healing specificity in target selection.

To further refine the therapeutic target selection, the 14 hub nodes were mapped against the four KEGG pathways relevant to wound healing to identify proteins with the most significant involvement throughout these pathways, as depicted in Figure 3b and Figure S1. Among the 14 hub nodes, five protein targets, IL6, FGF2, EGFR, KDR (VEGFR2), and INS, displayed the highest frequency of association across the four chosen pathways and were prioritized for subsequent molecular docking and in silico analysis. These targets were selected based on their well-established roles in wound healing physiology, including IL6 in mediating the transition from the inflammatory to proliferative phase via JAK/STAT3 pathway, FGF2 in promoting angiogenesis and fibroblast proliferation, EGFR in facilitating keratinocyte migration and re-epithelialization, VEGFR2 in driving VEGF-mediated angiogenesis, and insulin (INS) in regulating inflammatory responses and tissue repair metabolism [40,41]. Hub genes with high topological centrality but limited direct involvement in angiogenic or proliferative wound healing, including Caspase-3 (CASP3) and Tumor Protein p53 (TP53), which are associated with apoptotic regulation, and Albumin (ALB), which functions as a systemic plasma carrier protein, were absent from the four selected wound healing pathways and were excluded from further analysis.

### 2.4. Molecular Docking Analysis

Molecular docking serves as a reliable computational method for assessing ligand binding orientation, estimating binding affinity, and evaluating the stability of protein–ligand complexes, thereby providing important predictions regarding a compound’s potential efficacy as a pharmaceutical agent [42,43]. This method is well-suited for investigating phytochemicals as multi-targeted therapeutic candidates, as it enables simultaneous binding modes evaluation and interaction energy analysis across multiple protein targets. Accordingly, the binding properties of 18 phytochemicals extracted from *M. acuminata* peel were evaluated against five selected therapeutic targets: IL6 (PDB ID: 1ALU), FGF2 (PDB ID: 5X1O), EGFR (PDB ID: 5XWD), VEGFR2 (PDB ID: 6GQO), and INS (PDB ID: 6TC2) [44,45,46,47,48]. Prior to molecular docking, all proteins were subjected to preparation through energy minimization and 3D protonation to ensure structural integrity and optimal protonation states under simulated physiological conditions. The active site of each protein was identified using MOE Site Finder algorithm, with the cavity showing the highest propensity for ligand binding (PLB) score selected as the primary docking site (Supplementary Table S4).

**Figure 4:**
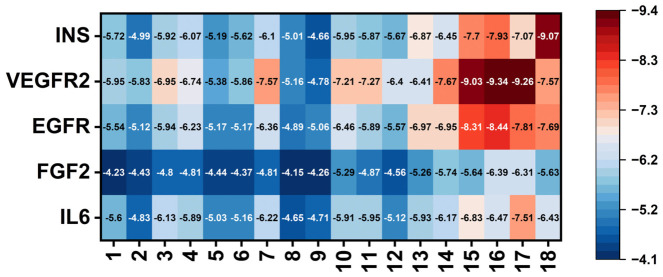
Molecular docking analysis of 18 selected phytochemicals against five key proteins associated with hemorrhagic wound healing. Binding energy values are expressed in kcal/mol.

Two complementary docking strategies, namely rigid docking and flexible docking, were applied. Rigid docking is based on the lock and key theory, which suggests that the protein binding pocket maintains a fixed conformation and only binds ligands with a structural geometry that is complementary to the binding site. In contrast, flexible docking functions by the induced fit theory, wherein the binding pocket undergoes conformational changes upon ligand interaction, accommodating a wider variety of ligands regardless of their initial structural configuration. Prior to docking simulation with the selected phytochemicals of *M. acuminata* peel, validation was conducted through re-docking of the co-crystallized native ligand within its binding site for each protein (Supplementary Figure S2). The results demonstrated RMSD values below 2 Å, confirming the reliability of the docking protocol [49]. The binding energy scores obtained from docking simulations of the 18 *M. acuminata* peel-derived phytochemicals against the five selected protein targets are depicted in Figure 4. Among the five protein targets, both FGF2 and IL6 exhibited consistently weak binding affinities throughout the majority of phytochemicals, ranging from −4.15 to −6.39 kcal/mol and −4.65 to −7.51, respectively, suggesting limited interaction potential with *M. acuminata* peel-derived phytochemicals. INS and EGFR showed weak to moderate binding affinities, ranging from −4.66 to −9.07 kcal/mol and −4.89 to −8.44 kcal/mol, respectively. In contrast, VEGFR2 showed the most favorable interactions across the phytochemical dataset, with Compounds **15**, **16**, and **17** exhibiting the lowest binding energies of −9.03, −9.34, and −9.26 kcal/mol, respectively, which indicates the most stable protein–ligand interactions.

To validate the docking results further, VEGFR2 (PDB ID: 6GQO) was additionally docked with Axitinib, an FDA-approved VEGFR2 inhibitor, as a positive reference control under identical docking conditions, resulting in a binding energy of −9.15 kcal/mol. Comparative analysis revealed that Compound **16** and Compound **17** demonstrated binding affinities superior to the Axitinib reference standard, while Compound **15** showed a lower binding affinity. Consequently, Compound **16** and Compound **17** further referred to as **BP16** and **BP17**, were selected as the lead compound candidates and prioritized for subsequent analysis to further characterize their interactions with VEGFR2. Each molecule exhibits a complex structure, comprising a fused pyrimido[5,4-d]pyrimidine and a piperazinyl acetamide, respectively. These are more generally associated with synthetic pharmaceutical compounds than plant-derived natural products. Although **BP16** and **BP17** were putatively annotated at MSI Level 2 based on MS2 spectral matching against the mzCloud database, the possibility of false-positive identification cannot be excluded due to the absence of reference standard confirmation [50]. However, their computational evaluation as lead scaffolds remains valid and meaningful within natural product-based drug discovery. The identified structural and pharmacophoric features provide a rational basis for the development of novel wound healing agents inspired by the chemical space of *M. acuminata* peel extract.

### 2.5. Molecular Interaction of ***BP16*** and ***BP17*** with VEGFR2

**Figure 5:**
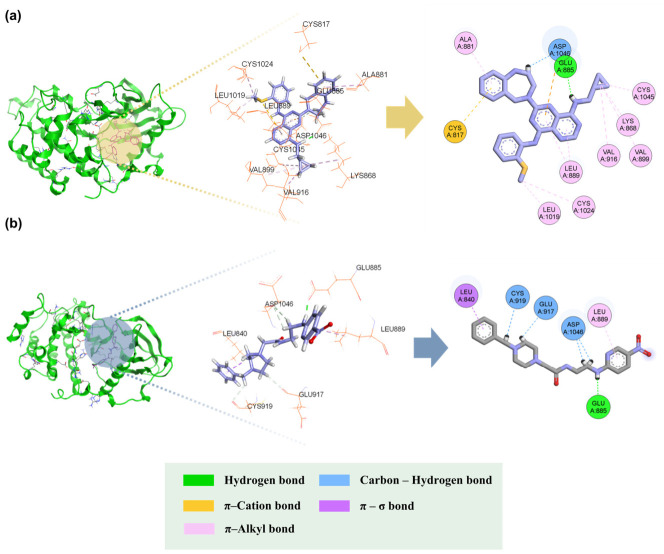
Molecular interaction between (**a**) **BP16** and (**b**) **BP17** with the VEGFR2 binding cavity.

Molecular docking simulation provides insight into the binding mode of small molecules within protein active sites, enabling the identification of key interacting amino acid residues and the characteristics of specific molecular interactions. In the case of **BP16** and **BP17** with VEGFR2, a broad range of non-covalent interactions formed within the protein–ligand complexes, involving conventional hydrogen bonds, carbon-hydrogen (C-H) bonds, and hydrophobic interactions including π-alkyl, π-cation, and π-σ interactions (Figure 5a,b, Table 2). Both compounds established conventional hydrogen bonds with GLU885 and similar C-H bonds with ASP1046, indicating a similar core binding mechanism within the VEGFR2 active site. These residues serve as critical components of VEGFR2 ATP-binding pocket, which consists of GLU917 and CYS919 constituting the hinge region, while GLU885 and ASP1046 contribute to the allosteric hydrophobic region [51].

Notably, **BP17** formed additional hydrogen bonds with GLU917 and CYS919, the key residues of the hinge region. This indicates more extensive engagement within the ATP-binding pocket relative to **BP16**, facilitated via stabilizing hydrogen bond interactions with GLU917 and CYS919 as the critical gatekeeper residues [52,53]. In contrast, **BP16** formed a more comprehensive network of hydrophobic interactions with residues LYS868, ALA881, LEU889, VAL899, VAL916, LEU1019, CYS1024, and CYS1045, which may collectively reflect in its slightly superior binding affinity relative to **BP17**, consistent with the role of hydrophobic interactions in stabilizing protein–ligand complexes.

To further evaluate the binding modes of **BP16** and **BP17**, the molecular interactions of Axitinib as a reference standard within the VEGFR2 binding site were characterized. As displayed in Supplementary Figure S2 and Table 2, Axitinib established various non-covalent interactions, including conventional hydrogen bonds, C-H bonds, and hydrophobic interactions, which consist of π-alkyl, alkyl-alkyl, and π-π stacking interactions. Notably, Axitinib formed hydrogen bonds with GLU885, GLU917, CYS919, and ASP1046, indicating a shared core binding mode with **BP16** and **BP17** at the VEGFR2 active site. With respect to hydrophobic interactions, Axitinib made contact with LEU840, VAL848, LYS868, LEU889, and VAL916, while PHE918 and PHE1047 participated in π-π stacking interactions with the compound. The shared key interacting residues between Axitinib and the two primary phytochemicals, especially within the ATP-binding pocket residues GLU917 and CYS919, suggest that both phytochemicals engage VEGFR2 through a binding mode consistent with that of known VEGFR2 ligands, supporting their ability to interact with this receptor. Previous study by Jain and Bhise (2024) revealed the computational wound healing effects of compounds such as gallic acid and quercetin on VEGFR2 [54]. This resulted in the formation of hydrogen bonds with the ATP-binding pocket residue GLU917, CYS919 and ASP1046, exhibiting binding affinities of −6.0 kcal/mol and −9.30 kcal/mol, respectively. Furthermore, Muftiasih et al. (2025) strengthened this finding by revealing comparable binding interactions of gallic acid and quercetin derived from *Spirulina platensis* with ATP-binding pocket of VEGFR2, displaying binding affinities of −6.01 kcal/mol and −9.21 kcal/mol, respectively [55]. Notably, **BP16** (−9.34 kcal/mol) and **BP17** (−9.26 kcal/mol) demonstrated binding affinities that are comparable or higher than those of gallic acid and quercetin, while interacting with the same essential residues, GLU917 and CYS919.

It is important to understand that molecular docking is unable to distinguish between agonistic and antagonistic mechanisms of receptor modulation. This approach quantifies binding affinity but cannot predict the functional result of ligand–receptor interaction. Furthermore, the complex formation of **BP16** and **BP17** with the VEGFR2 ATP-binding pocket through interactions with hinge region residues GLU917 and CYS919 does not provide mechanistic evidence of receptor activation. In standard kinase pharmacology, ATP-binding site occupancy is often associated with inhibition rather than activation, and the possibility that **BP16** and **BP17** act as VEGFR2 inhibitors cannot be excluded. VEGFR2 activation is physiologically favorable in wound healing by stimulating endogenous ligands like VEGF-A and FGF2 that activate downstream PI3K/AKT and MAPK signaling pathways, further facilitating endothelial cell proliferation, migration, and neovascularization essential to tissue repair [56,57]. However, the mechanistic role of **BP16** and **BP17** as potential VEGFR2 modulators remains to be experimentally determined through in vitro and in vivo assays.

### 2.6. Pharmacological Properties Prediction of ***BP16*** and ***BP17***

Pharmacokinetic evaluation was performed to assess the phytochemical’s predicted safety and efficacy through absorption, distribution, metabolism, excretion (ADME) and toxicity analysis. Based on the results shown in Table 3, both **BP16** and **BP17** demonstrated positive human intestinal absorption (HIA) prediction values of 97.9% and 94.5%, respectively, indicating favorable oral absorption through the human gastrointestinal tract. Considering the intended topical administration, the skin permeability for both compounds was also predicted. The predicted skin permeability coefficients (LogKp) of **BP16** (−2.74 cm/s) and **BP17** (−2.75 cm/s) indicate a moderate transdermal penetration potential. Moreover, as stated in Supplementary Table S3, both **BP16** and **BP17** displayed molecular weight of 484.63 Da and 398.465 Da, respectively, suggesting favorable passive diffusion through the stratum corneum [58]. Regarding lipophilicity, a critical parameter of partitioning between the stratum corneum and viable epidermis, **BP16** (LogP = 3.8405) showed a more favorable lipophilicity profiles for topical absorption compared to **BP17** (LogP = 0.0155) [59].

Regarding distribution, neither compound was predicted to pass through the blood–brain barrier (BBB), implying minimal likelihood of neurological side effects. The fraction unbound was higher for **BP17** than **BP16**, indicating a greater susceptibility for tissue distribution, including at the wound site, relative to plasma protein binding, which may enhance its local therapeutic availability [60].

Metabolic profiles of **BP16** and **BP17** were evaluated based on their affinity with cytochrome P450 (CYP) enzymes. **BP16** showed inhibitory activity against several CYP isoforms, whereas **BP17** exhibited no inhibitory effect except for CYP3A4, the predominant drug-metabolizing enzyme. Therefore, **BP16** presents a higher potential for drug–drug interactions [61]. The organic cation transporter 2 (OCT2) is a renal transport system responsible for the secretion of organic cations from blood plasma, such as dopamine, serotonin, and choline [62]. This finding implies favorable renal excretion properties for both phytochemicals, with no expected inhibition of OCT2-mediated transport. Additionally, toxicity prediction revealed that both **BP16** and **BP17** were predicted to be non-hepatotoxic, non-sensitizing to skin, and devoid of hERG I inhibitory activity, suggesting low toxicity to the human liver, skin, and cardiovascular system. However, **BP17** showed positive values for carcinogenicity and mutagenicity predictions. Taken together, toxicity prediction yielded **BP16** exhibiting a safer profile compared to **BP17**. Despite this, **BP17** was kept for further analysis due to the inherent limitations of computational toxicity predictions, which require experimental validation before achieving final conclusions (Table 4).

The drug-likeness and medicinal chemistry properties of **BP16** and **BP17** were evaluated to determine their suitability as potential drug candidates, with results described in Supplementary Table S5. Both compounds demonstrated bioavailability scores of 0.55, meeting the minimum accepted threshold for favorable oral bioavailability as defined by SwissADME [63]. Synthetic accessibility scores of 3.91 for **BP16** and 3.12 for **BP17** were reported, both within the range indicative of synthetically accessible compounds, showing that both candidates can be effectively synthesized by chemical synthesis. Both **BP16** and **BP17** demonstrated favorable drug-likeness scores of 4.734 and 2.105, respectively, suggesting their potential as candidates for hemorrhagic wound healing. The higher drug-likeness score of **BP16** indicates a more favorable molecular profile compared to **BP17**, corresponding with the higher toxicity profile observed in the previous analysis. The drug-likeness score is a composite metric that combines essential molecular properties such as lipophilicity, aqueous solubility, membrane permeability, metabolic stability, and binding affinity, thereby indicating the probability of a compound advancing through drug development pipelines.

### 2.7. Molecular Dynamics and MM/PBSA Calculation

**Figure 6:**
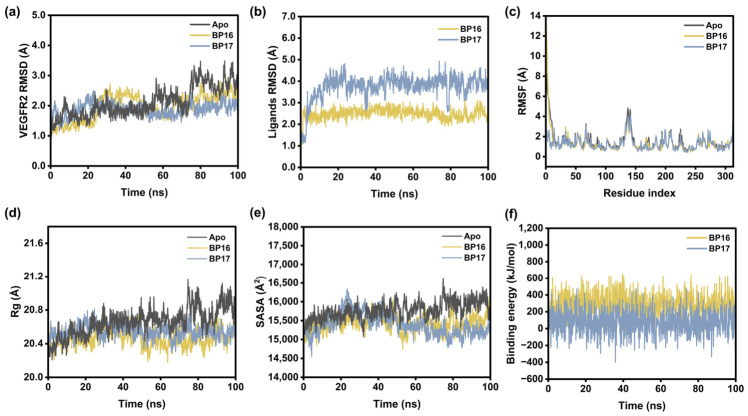
Molecular dynamics simulation over 100 ns. (**a**) Protein backbone RMSD, (**b**) ligand RMSD, (**c**) RMSF, (**d**) radius of gyration (Rg), (**e**) solvent accessible surface area (SASA), and (**f**) MM/PBSA analysis.

Molecular dynamics (MD) is an essential computational approach for evaluating the conformational stability of protein–ligand complexes under simulated physiological settings. In this study, MD simulations were employed over 100 ns to compare the stability of unbound VEGFR2 (apo-protein) with those of the VEGFR2-**BP16** and VEGFR2-**BP17** complexes. Analysis of protein RMSD revealed that both complexes displayed stable trajectories with minimal fluctuations, with average RMSD value of 1.963 ± 0.219 Å and 1.876 ± 0.439 Å for VEGFR2-**BP16** and VEGFR2-**BP17**, respectively (Figure 6a). In contrast, the apo-protein demonstrated higher RMSD fluctuations, averaging 2.113 ± 0.495 Å. This is consistent with the principle that proteins reduce their conformational flexibility upon ligand binding. Moreover, ligand binding induces conformational constraint via the formation of multiple non-covalent interactions, thermodynamically stabilizing the protein into a more defined conformational state [64]. Ligand RMSD analysis was subsequently performed to evaluate the structural stability of **BP16** and **BP17** within the VEGFR2 binding pocket throughout the simulation. As shown in Figure 6b, **BP16** possessed a lower average ligand RMSD of 2.479 ± 0.264 Å compared to **BP17** (3.743 ± 0.580 Å). Mann–Whitney U test confirmed that this difference was statistically significant (*p* < 0.0001), indicating the superior binding stability of **BP16** relative to **BP17** within the VEGFR2 binding pocket (Supplementary Table S6). This corresponds with the molecular docking result, wherein **BP16** exhibited a more favorable binding affinity with VEGFR2. To further characterize the conformational dynamics of the full protein structure, root mean square fluctuations (RMSF) analysis was performed to evaluate amino acid residue fluctuations in the apo-protein and both holoprotein complexes (VEGFR2-**BP16** and VEGFR2-**BP17**), as depicted in Figure 6c. All three systems showed comparable RMSF profiles overall, with high fluctuation observed at the N-terminal region, suggesting the inherent flexibility of terminal loop regions [65]. In contrast, residues within the active site exhibited consistently low fluctuations across the apo-protein and both holoproteins, indicating that ligand binding does not significantly impact the local conformational dynamics of the binding pocket residues.

The overall structural compactness and conformational integrity of the systems were analyzed through radius of gyration (Rg), as depicted in Figure 6d. All three systems showed comparable and stable Rg values throughout the simulation, with the apo-protein averaging 20.657 ± 0.161 Å, VEGFR2-**BP16** averaging 20.466 ± 0.101 Å, and VEGFR2-**BP17** averaging 20.545 ± 0.086 Å. The observed slight reduction in Rg for both holoprotein complexes compared to the apo-protein implies a slight compression of the protein structure upon ligand binding, which corresponds to the concept of ligand-induced conformational stabilization. Solvent accessible surface area (SASA) analysis was performed to assess the influence of solvent exposure on protein–ligand interaction. As shown in Figure 6e, all three systems exhibited stable SASA values, indicating consistent solvent exposure profiles throughout the simulation. Notably, both holoprotein complexes possessed lower average SASA values compared to the apo-protein (15,771.103 ± 236.783 Å^2^), with VEGFR2-**BP16** and VEGFR2-**BP17** averaging 15,424.568 ± 215.476 Å^2^ and 15,389.777 ± 285.731 Å^2^, respectively. The reduction in SASA upon ligand binding results from the burial of hydrophobic surface residues within the binding pocket, thus preserving them from solvent exposure and enhancing the thermodynamic stability of the complexes [66]. Binding free energy calculations via MM/PBSA were performed over the entire simulations to further validate the stability and affinity of the protein-ligand complexes (Figure 6f). Importantly, the MM/PBSA implementation in YASARA adopts an inverted sign standard relative to conventional MM/PBSA methods, wherein increasing positive binding free energy values signify more thermodynamic favorability of protein–ligand interactions [67,68,69]. Under this circumstance, VEGFR2-**BP16** showed a more favorable binding energy (287.878 ± 127.878 kJ/mol) compared to VEGFR2-**BP17** (71.442 ± 134.645 kJ/mol), consistent with the superior binding affinity observed in the molecular docking analysis. The relatively large standard deviations observed for both complexes correspond with the inherent dynamic behavior of protein–ligand interactions under physiological simulation, reflecting temporary conformational sampling induced by normal thermal movement rather than actual ligand dissociation events. This interpretation is corroborated by the stable ligand RMSD profiles of both complexes throughout the simulation period, reinforcing the stronger and more stable interaction of **BP16** within the VEGFR2 binding pocket. This is further supported by the stable ligand RMSD profiles for both complexes across the simulation time, which suggests a stronger and more constant interaction with **BP16** within the VEGFR2 binding pocket.

### 2.8. Chemical Reactivity and Electronic Properties Evaluation Using DFT Analysis

**Figure 7:**
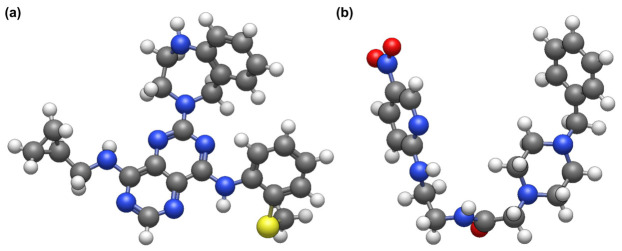
Optimized 3D geometries of (**a**) **BP16** and (**b**) **BP17**. Atom colors: carbon (grey), hydrogen (white), oxygen (red), nitrogen (blue), and sulfur (yellow).

The electronic properties and quantum mechanical reactivity of **BP16** and **BP17** were further characterized via DFT analysis. Evaluation of electronic properties enables prediction of metabolic behavior, safety profiles, and intermolecular charge transfer interactions, collectively providing insight into the intrinsic chemical reactivity of the candidate compounds [70]. Prior to electronic property evaluation, the geometries of **BP16** and **BP17** were optimized using the Becke, 3-parameter, Lee–Yang–Parr (B3LYP) hybrid functional. **BP16** exhibits a flat and rigid planar structure centered on a fused pyrimido[5,4-d]pyrimidine bicyclic aromatic core, consisting of three substituent arms: a 2-methylthiophenyl, a non-aromatic 1,4-benzodiazepine (BZD) ring, and a cyclopropylmethyl moiety, whose amine-containing groups serve as hydrogen bond donors, while the planar core facilitates hydrophobic interactions and π-stacking within the VEGFR2 binding pocket (Figure 7a). In contrast, **BP17** exhibits an amphiphilic character comprising three distinct structural domains: a lipophilic 1-benzylpiperazine arm, a planar glycylamide bridge, and a 2-amino-5-nitropyridine arm (Figure 7b). Despite their structural differences, both **BP16** and **BP17** possess lipophilic properties that make them susceptible to hydrophobic interactions and π-π stacking within the VEGFR2 binding pocket enhances their binding affinity for the receptor.

**Figure 8:**
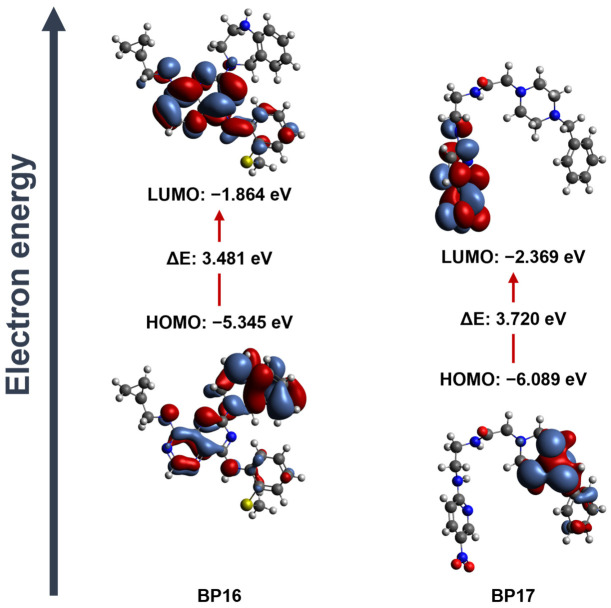
FMO analysis of **BP16** and **BP17**. Blue and red surfaces represent the positive and negative phases of the molecular orbital wavefunction, respectively.

The chemical structure of a phytochemical compound determines its electron distribution, which subsequently influences its reactivity and interaction potential. Based on the results of DFT calculations in Table 5, **BP16** displayed a lower total electronic energy than **BP17**, suggesting greater electronic stability and lower intrinsic reactivity, which may contribute to enhanced metabolic stability under physiological conditions. Moreover, the lower dipole moment of **BP16** relative to **BP17** suggests a more symmetrical electron distribution. This is due to extensive electron delocalization across the fused bicyclic pyrimidopyrimidine core and its conjugated BZD and methylthiophenyl ring system, resulting in a stronger electron-nuclear attraction and an overall more stable electronic configuration [71]. This is further reflected in the frontier molecular orbital (FMO) energy analysis of the highest occupied molecular orbital (HOMO) and the lowest unoccupied molecular orbital (LUMO), depicted in Figure 8. The HOMO-LUMO energy gap reflects molecular stability, representing the energy required for electron excitation from the highest occupied to the lowest unoccupied molecular orbital [72]. **BP16** demonstrated a slightly lower energy gap than **BP17**, suggesting marginally higher kinetic reactivity and stronger interaction with the binding pocket of VEGFR2. The higher HOMO and LUMO energies of **BP16** compared to **BP17** are further reflected in its lower ionization potential, electron affinity, and electronegativity. The extensive conjugated π-system resulting from the multiple fused aromatic rings of **BP16** significantly increases its HOMO energy. The lack of electron-withdrawing groups maintains an overall electron-rich electronic environment, which raises the LUMO energy and subsequently reduces both electron affinity and electrophilicity [73,74]. This signifies that **BP16** readily acts as an electron donor toward electron-acceptor residues in the binding site. These electronic properties suggest that **BP16** is less susceptible to electrophilic metabolism and nucleophilic attack under physiological conditions, potentially contributing to greater metabolic stability and a more favorable toxicity profile relative to **BP17**.

Complementary global reactivity descriptors, including chemical potential, molecular hardness, and molecular softness, were subsequently calculated to corroborate the electronic stability and reactivity trends observed from the HOMO-LUMO analysis (Table 6). The chemical stability of **BP16** was further supported by its molecular hardness and softness values, with **BP16** exhibiting lower chemical hardness and higher molecular softness compared to **BP17**, indicating a relatively soft nature of **BP16**. This result corresponds with its lower chemical potential, suggesting increased polarizability and enhanced capacity for charge transfer interactions of **BP16** within the VEGFR2 binding pocket [75].

Collectively, these findings provide a rational biological framework for the translational advancement of phytochemicals obtained from *M. acuminata* peel as natural therapeutic agents for wound healing. The identification of **BP16** and **BP17** as promising VEGFR2-interacting candidates, supported by their favorable ADMET profiles, underscores their physicochemical compatibility for integration into topical administration systems such as creams, hydrogels, or spray gels for the therapy of hemorrhagic wounds. In addition, *M. acuminata* peel is a sustainable and accessible agricultural by-product. Its utilization as a bioactive source corresponds with circular bioeconomy principles and provides a cost-effective alternative to synthetic wound healing agents. The comprehensive in silico pipeline employed in this study shows a scalable and resource-efficient scientific approach applicable to other underexplored medicinal plants. Thus, the above results support ongoing study of *M. acuminata* peel phytochemicals as potential lead candidates for hemorrhagic wound healing and offer logical molecular basis for subsequent validation via in vitro and in vivo analysis.

## 3. Materials and Methods

### 3.1. Musa acuminata Peels Preparation and Extraction

*Musa acuminata* (Cavendish bananas) were procured from a local market at Depok, West Java, Indonesia. The peels were separated from the fruits, rinsed thoroughly with distilled water to remove surface impurities, and subsequently dried in a conventional oven at 50 °C overnight. The dried peels were pulverized into fine powders using a grinder. Maceration was carried out by immersing the powder in ethyl acetate (≥99.5%, Merck, Germany) at a ratio of 1:7 (*w*/*v*) at room temperature under dark condition for 72 h. The extract was filtered with Whatman no. 1 filter paper, and the filtrate was concentrated using rotary evaporator (Eyela, Japan). The *M. acuminata* peel extract was stored at 4 °C for future use.

### 3.2. Phytochemical Profiling Using Liquid Chromatography and High-Resolution Mass Spectrometry (LC-HRMS/MS)

An untargeted metabolomics analysis approach was employed for phytochemical profiling of *M. acuminata* peel extract. Prior to analysis, 10 mg of banana peel extract was dissolved in 1 mL HPLC grade methanol, followed by vortexing and ultrasonication to ensure complete dissolution of the crude extract. The dissolved sample was filtered through a 0.2 µm nylon filter prior to injection. Chromatographic separation was performed utilizing a Thermo Scientific™ Vanquish™ Horizon UHPLC system equipped with a binary pump (Germering, Germany), coupled to a Thermo Scientific™ Orbitrap™ Exploris 240 high-resolution mass spectrometer (Bremen, Germany). Analyte separation was achieved using Thermo Scientific™ Accucore™ C18 column (100 mm length × 2.1 mm ID × 2.6 µm particle size; Lithuania) maintained at 40 °C. A sample volume of 5 µL was injected at a flow rate of 0.3 mL/min. The mobile phase consisted of MS grade water with 0.1% formic acid (Phase A) and MS grade acetonitrile with 0.1% formic acid (Phase B). Raw LC-HRMS data were analyzed using Compound Discoverer 3.3 software (Thermo Fisher Scientific, San Jose, CA, USA). Compound identification was performed through database matching with ChemSpider and Mass List for MS1 annotation, followed by MS2 spectral matching against the mzCloud database (Thermo Fisher Scientific, Waltham, MA, USA; accessed on 5 March 2025) for confirmatory annotation. In accordance with the Metabolomics Standards Initiative (MSI) reporting standards, all compound assignments are classified as putatively annotated metabolites at confidence Level 2.

### 3.3. In Silico Drug-Likeness Screening

The 3D structural coordinates of the phytochemicals identified from LC-HRMS analysis were obtained from PubChem (https://pubchem.ncbi.nlm.nih.gov/) in canonical SMILES format. Initial toxicological screening of candidate compounds was performed using OSIRIS DataWarrior software v06.01.00 to evaluate potential mutagenic, tumorigenic, irritant, and reproductive hazards profiles. In parallel, drug-likeness analysis was conducted by calculating key physicochemical descriptors, including total molecular weight, calculated partition coefficient (cLogP), number of hydrogen bond acceptors, number of hydrogen bond donors, topological polar surface area (TPSA), and number of rotatable bonds, based on Lipinski’s Rule of Five. Compounds fitting specified criteria were subsequently selected for further in silico analysis.

### 3.4. Potential Target and Network Pharmacology Construction

Putative protein targets of selected phytochemicals were predicted via SwissTargetPrediction web server (http://www.swisstargetprediction.ch/, accessed on 13 October 2025), with duplicate results subsequently deleted. Disease-related targets were obtained from the DisGeNET (https://disgenet.com/, accessed on 15 October 2025) and GeneCards (https://www.genecards.org/, accessed on 15 October 2025) databases, employing “open wound” and “hemorrhage” as search terms. Overlapping targets between the phytochemical target profiles and disease-related gene sets were identified through Venn diagram analysis using Venny 2.1.0 (https://bioinfogp.cnb.csic.es/tools/venny/, accessed on 15 October 2025). The intersecting targets were classified as the potential therapeutic targets for network pharmacology analysis. These targets were imported into the STRING database (https://cn.string-db.org/, accessed on 15 October 2025) with the organism set to “*Homo sapiens*” and a minimum interaction confidence threshold of 0.700. The resulting protein-protein interaction (PPI) data were imported into Cytoscape v3.10.4 software for network construction. To identify the core network, hub proteins were extracted based on three topological parameters: betweenness centrality (BC), degree centrality (DC) and closeness centrality (CC), with selection criteria defined as values exceeding the mean value of each parameter.

### 3.5. Gene Ontology Enrichment and Pathway Analysis

Gene Ontology (GO) enrichment analysis and Kyoto Encyclopedia of Genes and Genomes (KEGG) pathway enrichment analysis of the core target proteins were performed using the ShinyGO 0.85.1 web server (https://bioinformatics.sdstate.edu/go/, accessed on 9 November 2025), with a false discovery rate (FDR) cutoff of 0.05. The top 10 enriched GO terms and the top 10 KEGG pathways were selected for further analysis.

### 3.6. In Silico Screening via Molecular Docking Analysis

The 3D structures of the selected protein targets were downloaded from RSCB Protein Data Bank (http://www.rcsb.org/pdb, accessed on 24 November 2025). Protein preparation was done using the AMBER10:EHT force field with gas phase solvation. Protein optimization included hydrogen atom fixation and charge correction via the “Fix Hydrogen” and “Fix Charge” functions, followed by energy applied at an RMS gradient of 0.05 kcal/mol·Å. The binding site of each protein was defined by the co-crystallized native ligand and further validated using the MOE Site Finder algorithm. Ligand structures were optimized under the MMFF94x force field with gas phase solvation, followed by energy minimization with an RMS gradient of 0.001 kcal/mol/Å^2^. In this study, molecular docking simulation was performed in MOE 2019.01.02 software utilizing both rigid and flexible docking methods. The docking results were further ranked by their calculated binding free energy (∆G_binding_) values.

### 3.7. Pharmacokinetics and Toxicology Properties Prediction

Comprehensive pharmacokinetic profiling of the candidate ligands was conducted through prediction of ADME, toxicological, and medicinal chemistry properties. ADME properties were predicted using pkCSM and AdmetSAR 3.0. Toxicological assessments were carried out using ToxTree and ProTox 3.0 to evaluate potential safety liabilities of the candidate compounds. Medicinal chemistry properties were further assessed using OSIRIS DataWarrior and SwissADME.

### 3.8. Molecular Dynamics Simulation and Binding Free Energy Calculation

Molecular dynamics simulations were performed using YASARA (Yet Another Scientific Artifcial Reality Application) Dynamics software version 21.6.7 (Biosciences, Vienna, Austria) with the AMBER14 forcefield [76]. The simulation was configured at physiological condition of 25 °C, pH 7.4, 1 atm, and 0.9% NaCl for total simulation of 100 nanoseconds (ns). Post-simulation trajectory analysis was performed employing the md_analyze macro, which included root mean square deviation (RMSD), root mean square fluctuation (RMSF), hydrogen bond occupancy, radius of gyration, and solvent accessible surface area (SASA). Binding free energy was calculated over the entire 100 ns trajectory using the Molecular Mechanics Poisson–Boltzmann Surface Area (MM-PBSA) method via the md_analyzebindenergy macro. MD trajectories were further visualized and analyzed using BIOVIA Discovery Studio version 24.1 (Dassault Systèmes, Vélizy-Villacoublay, France).

### 3.9. Density Functional Theory

The electronic properties and molecular reactivity of the selected phytochemical candidates were evaluated using density functional theory (DFT) calculations conducted with ORCA version 5.0.4, while molecular visualization was carried out in Avogadro [77]. Prior to DFT calculations, geometry optimization and energy minimization were carried out employing the MMFF94 force field in the gas phase. Subsequent single-point DFT calculations were conducted utilizing the B3LYP hybrid functional to ascertain frontier molecular orbital (FMO) energies, including the highest occupied molecular orbital (HOMO), lowest unoccupied molecular orbital (LUMO), and the corresponding band gap energy. Furthermore, the FMO energies were employed to measure multiple quantum chemical reactivity descriptors, including chemical potential, hardness, and softness [78].

## 4. Conclusions

This study establishes an integrative in silico approach that combines LC-HRMS-based phytochemical profiling with comprehensive computational analysis, including network pharmacology, molecular docking, molecular dynamics simulations, and DFT, to identify the therapeutic potential of *Musa acuminata* peel ethyl acetate extract for hemorrhagic wound healing. Network pharmacology identified five key protein targets, IL6, FGF2, EGFR, KDR/VEGFR2, and INS, at the convergence of four wound healing-relevant signaling pathways: PI3K/AKT, MAPK, HIF-1, and Ras. Following Lipinski’s Rule of Five and toxicity screening, 18 phytochemical candidates were selected for molecular docking against these targets; **BP16** and **BP17** displayed the highest binding affinity for VEGFR2 (−9.34 and −9.26 kcal/mol, respectively). Through subsequent MD and DFT analysis, **BP16** showed better stability, favorable pharmacokinetic and toxicological profiles, and promising electronic properties. These results emphasize **BP16** as a promising lead compound and highlight the potential of *M. acuminata* peel as a previously unexplored source of bioactive compounds. This study offers a comprehensive computational approach aiming to accelerate drug discovery based on natural products for wound healing. Additional experimental validation is necessary to verify the therapeutic efficacy of the identified compounds.

## Figures and Tables

**Table 1:** LC-HRMS data of 18 *M. acuminata* peel-derived selected phytochemicals.

Compound No.	Peak No.	Compounds	Molecular Formula	Precursor Ion (*m*/*z*)	Adduct	MS/MS Fragments
**1**	22	Lucknolide A	C_10_H_12_O_6_	229.07048	[M + H]^+^	61.0283; 69.033; 97.028; 109.028; 127.039; 169.049; 229.069
**2**	70	Kynurenic acid	C_10_H_7_NO_3_	188.03543	[M − H]^−^	188.035
**3**	4	Inosine	C_10_H_12_N_4_O_5_	267.0722	[M − H]^−^	59.014; 71.014; 101.024; 131.035
**4**	26	4,6-(1′-carboxyethylidene)-3-O-methyl-β-D-glucopyranose	C_10_H_16_O_8_	309.08292	[M + FA − H]^−^	59.014; 71.014; 113.014
**5**	20	4-Formamido-1H-imidazole-5-sulfonamide	C_4_H_6_N_4_O_3_S	191.02251	[M + H]^+^	161.012; 191.023
**6**	67	Homoanatoxin A	C_11_H_17_NO	180.13803	[M + H]^+^	57.069; 79.054; 92.049; 93.069; 107.085; 135.117; 152.107; 163.111; 180.138
**7**	48	2,6,3′,4′-Tetrahydroxy-2-benzylcoumaranone	C_15_H_12_O_6_	287.0563	[M − H]^−^	83.014; 89.024; 125.024; 177.056; 243.067; 259.061; 287.057
**8**	7	(2R,3S)-2,3-Dimethylmalate	C_6_H_10_O_5_	161.04558	[M − H]^−^	43.018; 71.014; 73.029; 89.024; 97.029; 101.024
**9**	15	L-Threonic Acid	C_4_H_8_O_5_	135.02997	[M − H]^−^	134.865
**10**	23	5-Methoxy-N-[(3-methyl-1,2,4-oxadiazol-5-yl)methyl]-4-oxo-1,4-dihydro-2-pyridinecarboxamide	C_11_H_12_N_4_O_4_	299.05417	[M + Cl]^−^	59.014; 71.014; 113.025
**11**	55	4-(4-Methyl-5,7-dioxo-4,5-dihydro[1,2,5]thiadiazolo[3,4-d]pyrimidin-6(7H)-yl)butanoic acid	C_9_H_10_N_4_O_4_S	269.03452	[M − H]^−^	59.014; 167.003; 193.018; 213.008; 221.014; 241.039; 269.034
**12**	68	N-(2-Phenylethyl)-isobutyramide	C_12_H_17_NO	192.13799	[M + H]^+^	65.038; 72.044; 91.054; 100.075; 119.049; 192.138
**13**	148	7-Ketodeoxycholic acid	C_24_H_38_O_5_	429.26059	[M + Na]^+^	89.059
**14**	36	N~2~-[(4-Chloro-3,5-dimethyl-1H-pyrazol-1-yl)acetyl]-N-propylglycinamide	C_12_H_19_ClN_4_O_2_	285.11108	[M − H]^−^	123.045; 126.881; 183.913; 226.658
**15**	132	1-[(3R,9R,10R)-12-[(2S)-1-hydroxypropan-2-yl]-3,10-dimethyl-9-(methylaminomethyl)-13-oxo-2,8-dioxa-12-azabicyclo[12.4.0]octadeca-1(14),15,17-trien-16-yl]-3-propan-2-ylurea	C_26_H_44_N_4_O_5_	491.32263	[M − H]^−^	71.014; 283.265; 293.179; 297.279; 325.275; 339.199
**16**	51	N~8~-(Cyclopropylmethyl)-N~4~-[2-(methylsulfanyl)phenyl]-2-(1,2,3,5-tetrahydro-4H-1,4-benzodiazepin-4-yl)pyrimido[5,4-d]pyrimidine-4,8-diamine	C_26_H_28_N_8_S	483.20858	[M − H]^−^	61.082; 78.832; 90.619; 112.217; 125.089; 174.957; 226.659; 278.915
**17**	152	2-(4-Benzyl-1-piperazinyl)-N-{2-[(5-nitro-2-pyridinyl)amino]ethyl}acetamide	C_20_H_26_N_6_O_3_	399.21362	[M + H]^+^	109.064; 399.213
**18**	46	Cyclo-(L-Ile-L-Leu-L-Leu-L-Leu-L-Leu)	C_30_H_55_N_5_O_5_	588.40867	[M + Na]^+^	97.065; 114.091; 210.189; 227.175; 323.232; 340.259; 453.343; 548.417; 566.427

**Table 2:** Interacting amino acid residues of selected lead compounds and the standard ligand within the VEGFR2 binding site.

Compounds	Amino Acid Residues
**BP16**	CYS817, LYS868, ALA881, GLU885, LEU889, VAL899, VAL916, LEU1019, CYS1024, CYS1045, ASP1046
**BP17**	LEU840, GLU885, LEU889, GLU917, CYS919, ASP1046
**Axitinib**	LEU840, VAL848, ALA866, LYS868, GLU885, LEU889, VAL916, GLU917, PHE918, CYS919, LEU1035, ASP1046, PHE1047

**Table 3:** Predicted ADME properties of selected lead compounds derived from *M. acuminata* peel.

Compounds	Absorption		Distribution		Metabolism					Excretion
	^1^ HIA (%)	^2^ LogKp (cm/s)	^3^ BBB	^4^ *f*_u_	CYP1A2	CYP2C19	CYP2C9	CYP2D6	CYP3A4	^5^ OCT2
**BP16**	97.9	−2.74	−1.1	0.03	Yes	Yes	Yes	Yes	Yes	No
**BP17**	94.5	−2.75	−0.8	0.49	No	No	No	No	Yes	No

^1^ HIA: human intestinal absorption, ^2^ LogKp: skin permeability, ^3^ BBB: blood–brain barrier, ^4^ *f*_u_: fraction unbound, ^5^ OCT2: organic cation cransporter (SLC22A22).

**Table 4:** Predicted toxicological properties of *M. acuminata* peel-derived lead compounds **BP16** and **BP17**.

Compounds	LD_50_ (mg/kg)	Hepatotoxicity	Carcinogenicity	Mutagenicity	Skin Sensitization	hERG I Inhibitor
**BP16**	465	No	No	No	No	No
**BP17**	441	No	Yes	Yes	No	No

**Table 5:** DFT-calculated electronic properties of **BP16** and **BP17**.

Compounds	Electronic Energy (Hartree)	Dipole Moment (Debye)	Ionization Potential (eV)	Electron Affinity (eV)	Electronegativity (eV)
**BP16**	−1841.722	5.195	5.345	1.864	3.605
**BP17**	−1330.112	5.860	6.089	2.369	4.229

**Table 6:** Global reactivity descriptors of *M. acuminata* peel-derived lead compounds **BP16** and **BP17**.

Compounds	Chemical Potential (eV)	Hardness (eV)	Softness (eV)
**BP16**	3.6045	1.741	0.575
**BP17**	4.229	1.860	0.538

## Data Availability

The original contributions presented in this study are included in the article/Supplementary Material. Further inquiries can be directed to the corresponding author.

## Supplementary Materials

The following supporting information can be downloaded at: https://www.mdpi.com/article/10.3390/ph19070992/s1. Table S1: LC-HRMS-identified phytochemical constituents of the *M. acuminata* peel ethyl acetate extract; Table S2: Drug-likeness profiles of 18 *M. acuminata* peel-derived phytochemicals selected based on Lipinski’s Rule of Five screening; Table S3: Structures of 18 selected *M. acuminata* peel-derived phytochemicals; Table S4: Selected target proteins identified through network pharmacology analysis, including their 3D structures and predicted binding cavity residues; Table S5: In silico medicinal chemistry evaluation of *M. acuminata* peel-derived lead compounds **BP16** and **BP17**; Table S6: Statistical significance analysis of ligand RMSD values using the Mann-Whitney U test. Figure S1: Binary heatmap of target-pathway associations based on KEGG enrichment analysis of hemorrhagic wound healing-related pathways; Figure S2: Superimposition of the native ligand before (red) and after (blue) redocking within the binding sites of (a) IL6 (1ALU), (b) FGF2 (5X1O), (c) EGFR (5XWD), (d) VEGFR2 (6GQO), and (e) INS (6TC2); Figure S3: Molecular interactions of Axitinib with key residues in the VEGFR2 binding cavity.

## Abbreviations

The following abbreviations are used in this manuscript:

ADME	Absorption, Distribution, Metabolism, and Excretion
AgNPs	Silver Nanoparticles
AKT	Protein Kinase B
AMBER	Assisted Model Building with Energy Refinement
ATP	Adenosine Triphosphate
BBB	Blood-Brain Barrier
BC	Betweenness Centrality
B3LYP	Becke, 3-Parameter, Lee-Yang-Parr hybrid functional
BZD	Benzodiazepine
CC	Closeness Centrality
cLogP	Calculated Partition Coefficient
COX-1	Cyclooxygenase-1
CYP	Cytochrome P450
DC	Degree Centrality
DFT	Density Functional Theory
ECM	Extracellular Matrix
EGFR	Density Functional Theory
FDR	False Discovery Rate
FGF2	Fibroblast Growth Factor 2
FMO	Frontier Molecular Orbital
GO	Gene Ontology
HIA	Human Intestinal Absorption
HIF-1	Hypoxia-Inducible Factor 1
HOMO	Highest Occupied Molecular Orbital
IL6	Interleukin-6
INS	Insulin
KEGG	Kyoto Encyclopedia of Genes and Genomes
KDR	Kinase Insert Domain Receptor
LC-HRMS	Liquid Chromatography–High Resolution Mass Spectrometry
LUMO	Lowest Unoccupied Molecular Orbital
MAPK	Mitogen-Activated Protein Kinase
MD	Molecular Dynamics
MM/PBSA	Molecular Mechanics Poisson–Boltzmann Surface Area
MMFF94x	Merck Molecular Force Field 94x
MOE	Molecular Operating Environment
ns	Nanoseconds
OCT2	Organic Cation Transporter 2
PDB	Protein Data Bank
PI3K	Phosphoinositide 3-Kinase
PLB	Propensity for Ligand Binding
PPI	Protein–Protein Interaction
Ras	Rat Sarcoma
Rg	Radius of Gyration
RMSD	Root Mean Square Deviation
RMSF	Root Mean Square Fluctuation
ROS	Reactive Oxygen Species
SASA	Solvent Accessible Surface Area
SMILES	Simplified Molecular Input Line Entry System
STAT3	Signal Transducer and Activator of Transcription 3
STRING	Search Tool for the Retrieval of Interacting Genes/Proteins
TNF-α	Tumor Necrosis Factor Alpha
TPSA	Topological Polar Surface Area
UHPLC	Ultra-High Performance Liquid Chromatography
