# Supplementary material for: Phytochemical Profiling and Computational Screening of Musa acuminata Peel as Hemorrhagic Wound Treatment Candidate: Network Pharmacology, Molecular Docking, Molecular Dynamics, and DFT Approaches
Source: Pharmaceuticals (Basel). 2026 Jun 26;19(7):992. doi: 10.3390/ph19070992 (PMC13415815; doi:10.3390/ph19070992)
Supplement: Supplementary file 1 [file pharmaceuticals-19-00992-s001.zip › 2. Fixed Manuscript SI_ver 1.0.pdf]

Supporting Information

Phytochemical Profiling and Computational Screening of *Musa acuminata* Peel as Hemorrhagic Wound Treatment Candidate: Network Pharmacology, Molecular Docking, Molecular Dynamics, and DFT Approaches

Andi Darma Putra[1,2,3*], Naufal Syafiq Darmawan[2,3], Lasmini Syariatin[2,3], Aldi Tamara Rahman[2,3], Edwin Jeika Bunggulawa[4], Firda Puspita[2,3]

[1]Division of Gynecologic Oncology, Department of Obstetrics and Gynecology, Faculty of Medicine, Universitas Indonesia, Cipto Mangunkusumo Hospital, Central Jakarta 10430, Indonesia.

[2]Ovarian, Tubal, and Peritoneal Malignancy Research Unit, Department of Obstetrics and Gynecology, Faculty of Medicine, Universitas Indonesia, Cipto Mangunkusumo Hospital, Central Jakarta 10430, Indonesia.

[3]Dopamine Science Institute, Pancoran Mas, Depok 16431, Indonesia.

[4]Department of Pharmacy, Faculty of Health Sciences, Universitas Pelita Harapan, Tangerang 15811, Indonesia

*Correspondence: andi.darma@ui.ac.id; Tel.: +62-8161457653

Table S2. Drug-likeness profiles of 18 *M. acuminata* peel-derived phytochemicals selected based on Lipinski's Rule of Five screening.

Compound No.	Compounds	Molecular weight (<500 Da)	cLogP (≤5)	H-bond donor (≤5)	H- bond acceptor (≤10)	Rotatable bonds (≤10)
**1**	Lucknolide A	228.199	-1.5928	3	6	1
**2**	Kynurenic acid	189.17	0.2516	2	4	1
**3**	Inosine	268.228	-2.266	4	9	2
**4**	4,6-(1′-carboxyethylidene)-3-O-methyl-β-D-glucopyranose	264.229	-2.2123	3	8	2
**5**	4-Formamido-1H-imidazole-5-sulfonamide	190.183	-1.6345	3	7	2
**6**	Homoanatoxin A	179.262	1.4633	1	2	2
**7**	2,6,3',4'-Tetrahydroxy-2-benzylcoumaranone	288.254	1.6532	4	6	2
**8**	(2R,3S)-2,3-Dimethylmalate	160.125	-5.2415	1	5	3
**9**	L-Threonic Acid	136.103	-2.3606	4	5	3
**10**	5-Methoxy-N-[(3-methyl-1,2,4-oxadiazol-5-yl)methyl]-4-oxo-1,4-dihydro-2-pyridinecarboxamide	264.24	-1.3967	2	8	4
**11**	4-(4-Methyl-5,7-dioxo-4,5-dihydro[1,2,5]thiadiazolo[3,4-d]pyrimidin-6(7H)-yl)butanoic acid	270.268	0.6446	1	8	4
**12**	N-(2-Phenylethyl)-isobutyramide	191.273	2.1755	1	2	4
**13**	7-Ketodeoxycholic acid	406.561	3.3231	3	5	4
**14**	N~2~-[(4-Chloro-3,5-dimethyl-1H-pyrazol-1-yl)acetyl]-N-propylglycinamide	286.762	0.0015	2	6	6
**15**	1-[(3R,9R,10R)-12-[(2S)-1-hydroxypropan-2-yl]-3,10-dimethyl-9-(methylaminomethyl)-13-oxo-2,8-dioxa-12-azabicyclo[12.4.0]octadeca-1(14),15,17-trien-16-yl]-3-propan-2-ylurea	492.658	2.8565	4	9	6
**16**	N~8~-(Cyclopropylmethyl)-N~4~-[2-(methylsulfanyl)phenyl]-2-	484.63	3.8405	3	8	7

	(1,2,3,5-tetrahydro-4H-1,4-benzodiazepin-4-yl)pyrimido[5,4-d]pyrimidine-4,8-diamine					
**17**	2-(4-Benzyl-1-piperazinyl)-N-{2-[(5-nitro-2-pyridinyl)amino]ethyl}acetamide	398.465	0.0155	2	8	9
**18**	Cyclo-(L-Ile-L-Leu-L-Leu-L-Leu-L-Leu)	565.797	2.8505	5	10	10

Table S3. Structures of 18 selected *M. acuminata* peel-derived phytochemicals.

Compound No.	Compounds	PubChem ID	SMILES	Molecular Structures
**1**	Lucknolide A	46933683	C1=C[C@@H]2[C@@H]3[C@H]1[C@H](O[C@@H]3[C@@](OC2=O)(CO)O)O	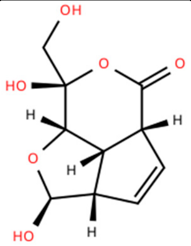
**2**	Kynurenic acid	3845	C1=CC=C2C(=C1)C(=O)C=C(N2)C(=O)O	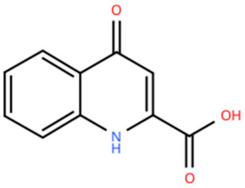
**3**	Inosine	135398641	C1=NC2=C(C(=O)N1)N=CN2[C@H]3[C@@H]([C@@H]([C@H](O3)CO)O)O	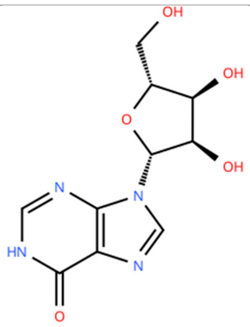
**4**	4,6-(1′-carboxyethylidene)-3-O-methyl-β-D-glucopyranose	45480536	C[C@@]1(OC[C@@H]2[C@@H](O1)[C@@H]([C@H]([C@@H](O2)O)O)OC)C(=O)O	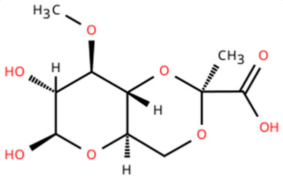
**5**	4-Formamido-1H-imidazole-5-sulfonamide	12585379	C1=NC(=C(N1)S(=O)(=O)N)NC=O	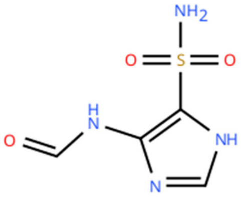
**6**	Homoanatoxin A	126727	CCC(=O)C1=CCC[C@@H]2CC[C@H]1N2	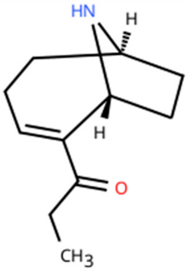

**7**	2,6,3',4'-Tetrahydroxy-2-benzylcoumaranone	42607788	C1=CC(=C(C=C1CC2(C(=O)C3=C(O2)C=C(C=C3)O)O)O)O	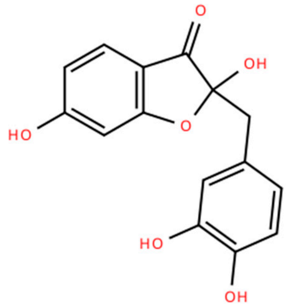
**8**	(2R,3S)-2,3-Dimethylmalate	25200436	C[C@H](C(=O)[O-])[C@@](C)(C(=O)[O-])O	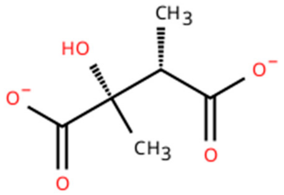
**9**	L-Threonic Acid	5460407	C([C@@H]([C@H](C(=O)O)O)O)O	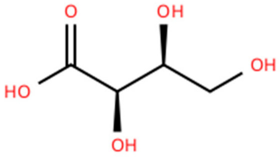
**10**	5-Methoxy-N-[(3-methyl-1,2,4-oxadiazol-5-yl)methyl]-4-oxo-1,4-dihydro-2-pyridinecarboxamide	127254661	CC1=NOC(=N1)CNC(=O)C2=CC(=O)C(=CN2)OC	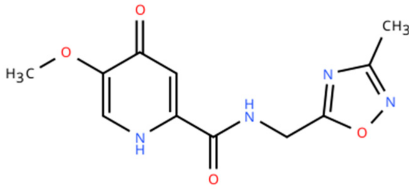
**11**	4-(4-Methyl-5,7-dioxo-4,5-dihydro[1,2,5]thiadiazolo[3,4-d]pyrimidin-6(7H)-yl)butanoic acid	10539960	CN1C2=NSN=C2C(=O)N(C1=O)CCCC(=O)O	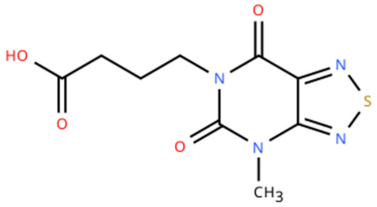
**12**	N-(2-Phenylethyl)-isobutyramide	290550	CC(C)C(=O)NCCC1=CC=CC=C1	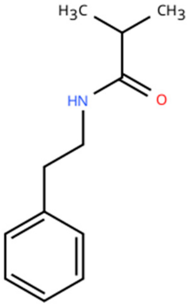

13	7-Ketodeoxycholic acid	188292	C[C@H](CCC(=O)O)[C@H]1CC[C@@H]2[C@@]1([C@H](C[C@H]3[C@H]2C(=O)C[C@H]4[C@@]3(CC[C@H](C4)O)C)O)C	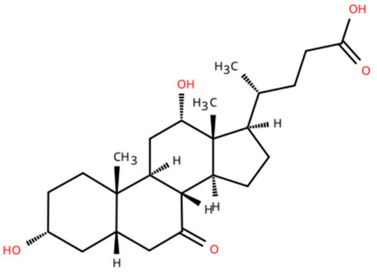
14	N~2~-[(4-Chloro-3,5-dimethyl-1H-pyrazol-1-yl)acetyl]-N-propylglycinamide	71938717	CCCNC(=O)CNC(=O)CN1C(=C(C(=N1)C)Cl)C	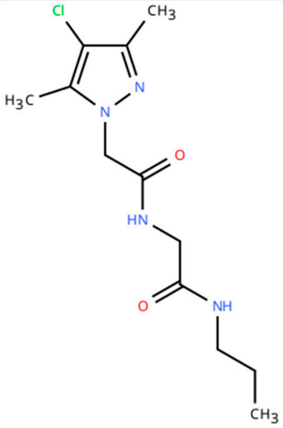
15	1-[(3R,9R,10R)-12-[(2S)-1-hydroxypropan-2-yl]-3,10-dimethyl-9-(methylaminomethyl)-13-oxo-2,8-dioxa-12-azabicyclo[12.4.0]octadeca-1(14),15,17-trien-16-yl]-3-propan-2-ylurea	44495045	C[C@@H]1CCCCO[C@H]([C@@H](CN(C(=O)C2=C(O1)C=CC(=C2)NC(=O)NC(C)C)[C@@H](C)CO)C)CNC	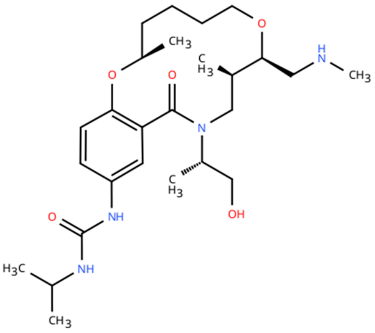
16	N~8~-(Cyclopropylmethyl)-N~4~-[2-(methylsulfanyl)phenyl]-2-(1,2,3,5-tetrahydro-4H-1,4-benzodiazepin-4-yl)pyrimido[5,4-d]pyrimidine-4,8-diamine	66552424	CSC1=CC=CC=C1NC2=NC(=NC3=C2N=CN=C3NCC4CC4)N5CCNC6=CC=CC=C6C5	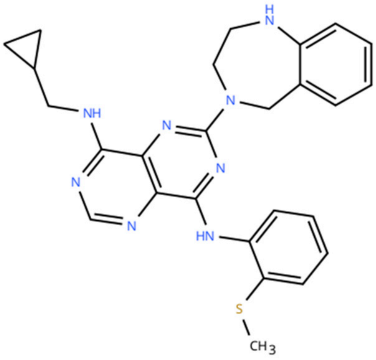
17	2-(4-Benzyl-1-piperazinyl)-N-{2-[(5-nitro-2-pyridinyl)amino]ethyl}acetamide	4976666	C1CN(CCN1CC2=CC=CC=C2)CC(=O)NCCNC3=NC=C(C=C3)[N+](=O)[O-]	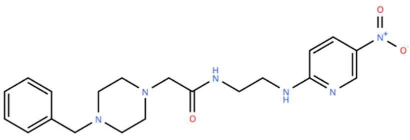

18	Cyclo-(L-Ile-L-Leu-L-Leu-L-Leu-L-Leu)	102342363	CC[C@H](C)[C@H]1C(=O)N[C@H](C(=O)N[C@H](C(=O)N[C@H](C(=O)N[C@H](C(=O)N1)CC(C)C)CC(C)C)CC(C)C)CC(C)C	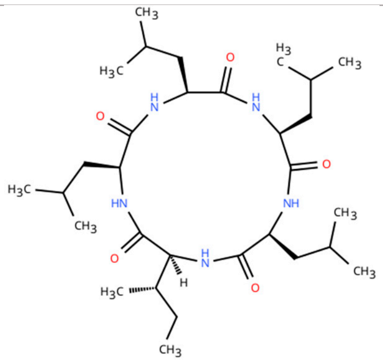

Table S4. Selected target proteins identified through network pharmacology analysis, including their 3D structures and predicted binding cavity residues.

Target Proteins	3D Structure	Binding Cavity Residues
IL6 (PDB ID: 1ALU)	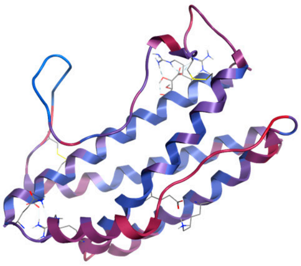	GLU42, THR43, LYS46, SER47, TYR100, ASN103, ARG104, PHE105, GLU106, SER107, SER108, GLN152, GLN156, ASP160, THR163
FGF2 (PDB ID:5X1O)	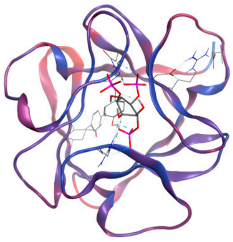	GLN54, LEU55, GLN56, GLU58, SER64, LYS66, TYR73, VAL88, PHE93
EGFR (PDB ID: 5XWD)	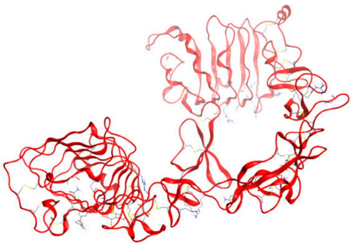	LYS375, GLU397, GLU400, ARG427, SER428, LEU329, LYS430, LYS455, LEU456, TRP492, PRO496, ARG497, ASP498, CYS499, VAL500, SER501, CYS502, SER506, GLY508, ARG509, GLU510, CYS511, GLU530
VEGFR2 (PDB ID: 6GQO)	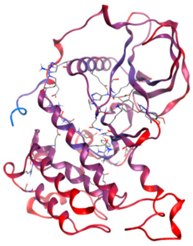	ASP814, CYS817, LEU840, VAL848, ALA866, VAL867, LYS868, ALA881, LEU882, SER884, GLU885, ILE888, LEU889, ILE892, VAL898, VAL899, VAL914, VAL916, GLU917, PHE918, CYS919, LYS920, PHE921, GLY922, ASN923, LEU1019, CYS1024, ILE1025, HIS1026, ARG1027, ASP1028, LEU1035, ILE1044, CYS1045, ASP1046, PHE1047, GLY1048, LEU1049, ARG1051, ILE1053, TYR1054, TYR1059, ARG1066, LEU1067, PRO1068, MET1072, THR1076, TYR1082
INS (PDB ID: 6TC2)	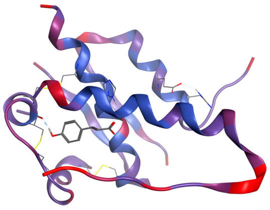	1:(VAL2, HIS5, LEU6); 3:(CYS6, SER9, ILE10, CYS11, SER12, LEU13, LEU16): 4:(CYS7, HIS10, LEU11, ALA14, LEU17, VAL18)

Table S5. In silico medicinal chemistry evaluation of *M. acuminata* peel-derived lead compounds BP16 and BP17.

Compounds	Bioavailability	Synthetic Accessibility	Drug-likeness
BP16	0.55	3.91	4.734
BP17	0.55	3.12	2.105

Table S6. Statistical significance analysis of ligand RMSD values using the Mann–Whitney U test.

Complex	Mean RMSD (Å)	SD (Å)	*p*-value
VEGFR2-BP16	2.479	0.264	< 0.0001
VEGFR2-BP17	3.743	0.580	

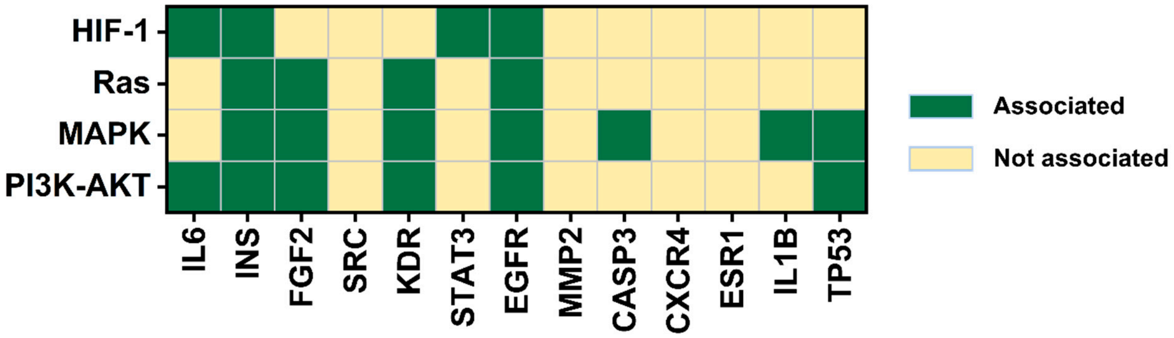

Figure S1. Binary heatmap of target–pathway associations based on KEGG enrichment analysis of hemorrhagic wound healing–related pathways.

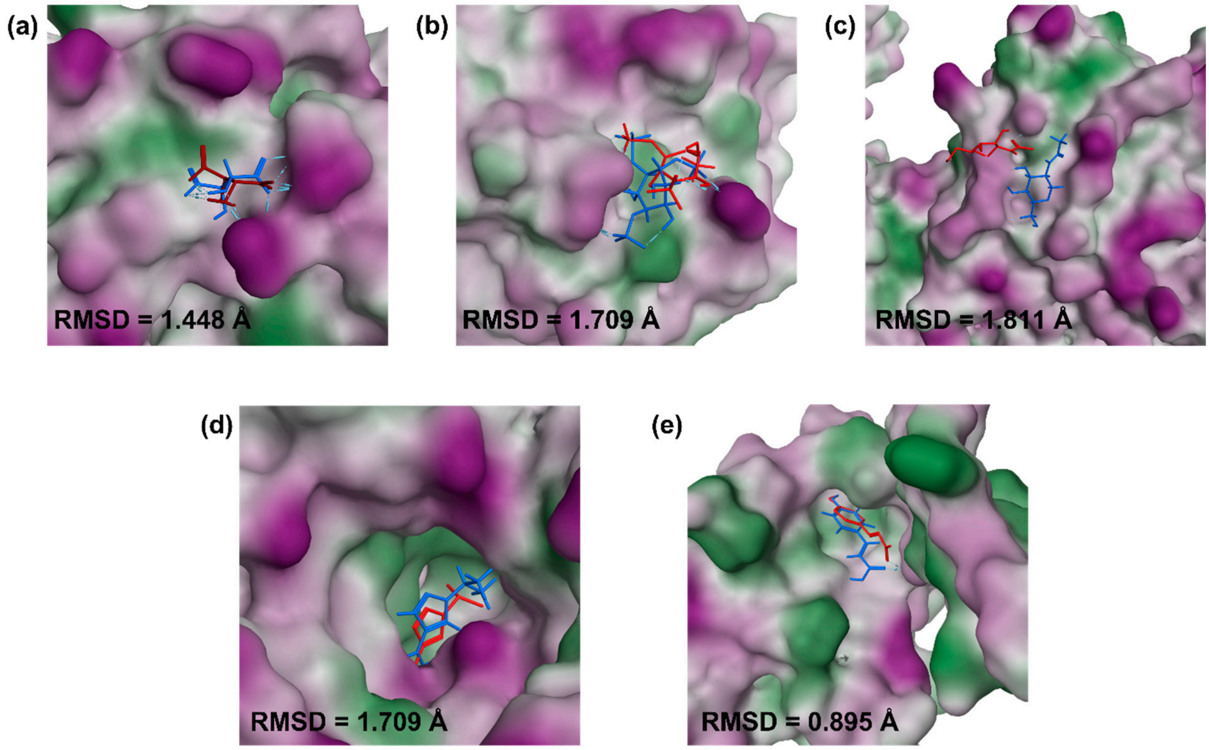

Figure S2. Superimposition of the native ligand before (red) and after (blue) redocking within the binding sites of (a) IL6 (1ALU), (b) FGF2 (5X1O), (c) EGFR (5XWD), (d) VEGFR2 (6GQO), and (e) INS (6TC2).

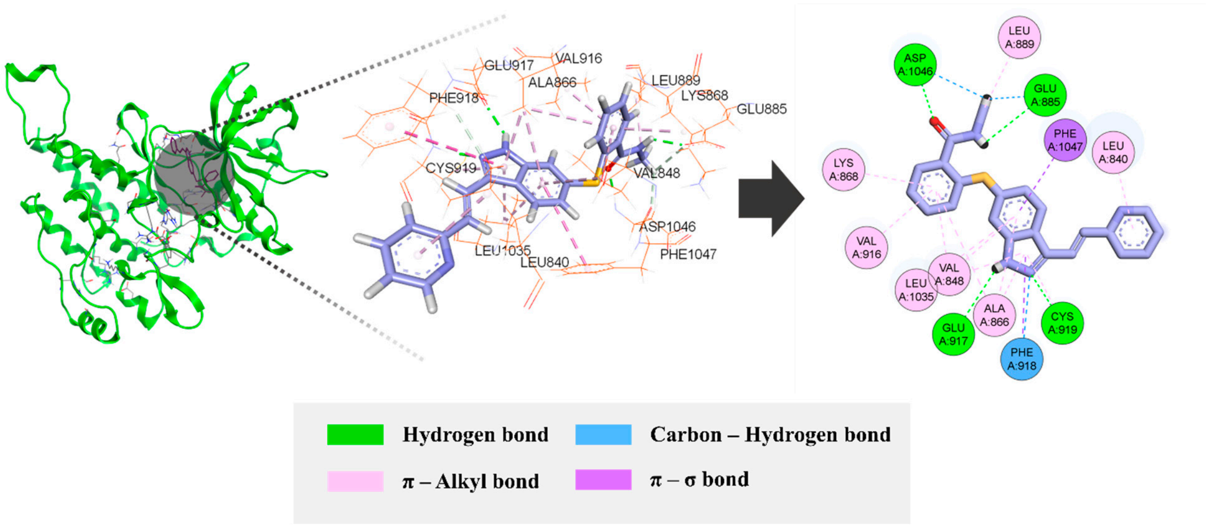

Figure S3. Molecular interactions of Axitinib with key residues in the VEGFR2 binding cavity.
